# Anterior segment alterations in fibromyalgia syndrome: a cross-sectional study on dry eye disease, meibomian gland dysfunction, and astigmatism

**DOI:** 10.1007/s00296-025-05872-w

**Published:** 2025-04-25

**Authors:** Duygu Topaktaş Emekli, Eda Sahutoglu, Gülşah Yaşa Öztürk, Burhan Fatih Kocyigit

**Affiliations:** 1 Department of Ophthalmology, Adana City Training and Research Hospital, Adana, Türkiye; 2 Faculty of Medicine, Department of Physical Medicine and Rehabilitation, University of Health Sciences, Adana City Training and Research Hospital, Adana, Türkiye

**Keywords:** Fibromyalgia, Ophthalmology, Eye diseases, Dry eye disease, Astigmatism, Meibomian gland dysfunction

## Abstract

Fibromyalgia syndrome (FMS) is an ongoing disorder distinguished by its multifaceted characteristics. There has been increasing interest in its ophthalmologic aspects. This study evaluated the anterior segment parameters of the eye in patients with FMS, comparing them to healthy controls. Furthermore, correlations with disease activity were analyzed. This research was structured as a cross-sectional and comparative analysis. Data was collected at a tertiary care education and research hospital from August 20, 2024, to November 15, 2024. The participants’ central corneal thickness, uncorrected and best-corrected visual acuities, refraction parameters, intraocular pressure, meibomian gland dysfunction (MGD), and dry eye disease (DED) parameters were evaluated. There were no significant differences in visual acuity, spherical equivalent, intraocular pressure, and central corneal thickness between FMS and healthy controls (*p* > 0.05). The median astigmatism in the right eye (diopter) was − 0.50 (-8.25–0) for the FMS group and − 0.12 (-1.75–0.50) for the control group (*p* = 0.012). The left eye data were − 0.50 (-2–1.50) for the FMS group and 0 (-1.75–0.75) for the control group (*p* = 0.056). MGD was identified in 26 (36.6%) individuals in the FMS group, whereas it was identified in 3 (4.3%) participants in the control group (*p* < 0.001). DED was detected in 43 (60.6%) participants in the FMS group and 15 (21.4%) participants in the control group (*p* < 0.001). FMS patients had higher rates of astigmatism, MGD, and DED compared to healthy controls, underscoring the necessity for routine anterior segment assessments. These data suggest possible ocular involvement in FMS, which warrants further research.

**Supplementary Information:**

The online version contains supplementary material available at 10.1007/s00296-025-05872-w.

## Introduction

Fibromyalgia syndrome (FMS) is a chronic condition that is characterized by widespread pain in the musculoskeletal system, tiredness, sleep difficulties, and abnormalities in mental abilities [1, 2]. Women are more likely to be affected by FMS, which is defined as a complicated pathophysiology that includes central sensitization, neurotransmitter abnormalities, and autonomic dysfunction of the nervous system. The precise etiology of FMS is unidentified; nonetheless, its manifestations frequently result in a considerable decline in quality of life and physical capability [3, 4]. Besides its characteristic symptoms, FMS can be linked to various comorbidities, including emotional disorders, irritable bowel syndrome, and headaches, illustrating its multifaceted nature [5, 6].

In recent years, there has been a growing interest in the ophthalmologic involvement of FMS as research has begun to reveal ocular manifestations in these patients [7]. Among the most common complaints are dry eye symptoms, photophobia, and vision problems, all of which are hypothesized to be associated with immunological alterations and autonomic nervous system dysfunctions [8, 9]. The researchers identified decreased ocular sensitivity in individuals with FMS, attributing this to a persistent reduction in tear production [10]. Patients with chronic pain syndrome have dry eye symptoms, even when their clinical grade of ocular surface illness is comparable to or less severe than that of dry eye patients without chronic pain [11]. Ocular manifestations may contribute to the overall burden of the disease; nevertheless, the comprehensive level of ocular involvement in FMS is still not completely understood.

In a prior investigation, we assessed the posterior segment of the eye in individuals with FMS, concentrating on retinal and optic nerve measures [12]. The current study assesses the anterior segment of the eye in individuals with FMS. We particularly compare visual acuity, astigmatism, axis, intraocular pressure, central corneal thickness, meibomian gland dysfunction, dry eye disease, and the severity of dry eye between FMS patients and healthy controls. Additionally, we intend to investigate the correlations between FMS disease activity and these ocular parameters to enhance our comprehension of the link between FMS and ophthalmic health.

## Methods

This study was designed as a cross-sectional and comparative analysis. Data was obtained at a tertiary care education and research hospital’s Physical Medicine and Rehabilitation and Ophthalmology clinics. The data-collecting period spanned from August 20, 2024, to November 15, 2024. Inclusion in the FMS group was contingent upon fulfilling the 2016 American College of Rheumatology (ACR) criteria [13]. The research comprised participants aged 18 and above, with healthy controls matched to the baseline features of the FMS group. Participants newly diagnosed with FMS were included in the research to prevent the influence of FMS-related medication on results. All subjects had an ophthalmological evaluation of the posterior segment, and situations with abnormalities were excluded. The exclusion criteria were as follows: non-compliant ophthalmologic examination, inflammatory rheumatic-autoimmune diseases, multiple sclerosis, and neurologic disorders potentially influencing ocular findings, history of ocular surgery or trauma, uveitis, diabetes mellitus, cardiac disorders, vascular system diseases, hypertension, retinal diseases, glaucoma, maculopathy, keratopathy, lacrimal duct stenosis and taking drugs that may affect results. Patients were questioned verbally on all these exclusion criteria. The Ministry of Health registration system was reviewed for previous diagnoses and medications following the acquisition of patient permission. All subjects underwent standardized laboratory examinations, and those with any results indicating potential violations of the exclusion criteria were excluded.

The case and control groups recorded their ages, genders, body mass indexes (BMIs), the duration of symptoms, occupational positions, and educational levels. The same physical medicine and rehabilitation specialist validated the diagnosis of FMS and its related clinical consequences. A single ophthalmologist performed all ophthalmological evaluations with 15 years of experience in the field. To reduce bias, the ophthalmologist was unaware of the participants’ group assignments (FMS vs. controls) during the assessments. Additionally, an independent researcher distributed the questionnaire forms before the ophthalmological examination, ensuring that the examiner did not impact the participants’ responses.

### Fibromyalgia impact questionnaire

The FIQ is an instrument that offers detailed questions designed to evaluate and analyze the various implications of FMS on individuals’ daily lives and routines. The FIQ was initially created by Burckhardt et al. [14] and has since become an extensively utilized and established measure for evaluating FMS severity in clinical and research contexts. The Turkish version of this instrument has demonstrated reliability and validity [15]. The questionnaire has 10 fundamental questions. A score of 100 is the maximum attainable score, with higher numbers indicating greater severity of participation.

### Ophthalmologic evaluation

The participants’ central corneal thickness (CCT) was measured using the Nidek UP-1000 ultrasonic pachymetry equipment. The central corneal thickness was the average of three consecutive measurements performed centrally in eyes without pupil dilation. Refraction parameters (spherical equivalent, astigmatism, and axis) were measured using an auto refractometer (Topcon KR-800 Auto Kerato-Refractometer, Tokyo, Japan).

Uncorrected and best-corrected visual acuities were determined using Snellen’s chart. Anterior segment structures were evaluated by slit lamp biomicroscopy (Topcon SL-3G Slit Lamp, Tokyo, Japan), and dilated fundus was examined with a 90D lens. Intraocular pressure was measured with Goldman applanation tonometer (Haag-Streit, USA) after topical anesthetic drops and corneal staining with fluorescein (Haag-Streit).

Thickening or irregularity of the eyelid margin, erythema on the posterior margin of the eyelid, telangiectasia, dilatation of blood vessels around the meibomian glands, very dense turbid, foamy and viscous secretion coming from the gland orifices with digital pressure and dilatation of the orifices of the meibomian glands were used as diagnostic criteria for meibomian gland dysfunction (MGD) in biomicroscopic examination [16, 17].

The ocular surface staining test was used to diagnose dry eye, so fluorescein stain was dripped onto the ocular surface. The ocular surface staining patterns were then examined with the microscope’s blue light filter, and the severity of the disease was recorded according to the Oxford Staining Scale. Keratoconjunctival staining was graded as mild (stages 0 or 1), moderate (stages 2 or 3), or severe (stages 4 or 5) [18, 19].

The local Research Ethics Committee granted clearance for this study following a comprehensive assessment of the ethical factors (approval date: August 15, 2024; approval number: 96). Written consent was obtained from all participants. The Helsinki standards were adhered to rigorously throughout the examination.

### Statistical analysis

Data analysis was conducted utilizing the Statistical Package for the Social Sciences (SPSS) version 23 software. The results were presented as numbers, percentages, and median (minimum-maximum). The determination regarding the conformity of the data to a normal distribution was founded on the outcomes of the Shapiro-Wilk test. Continuous variables were compared across groups through the Mann-Whitney U-test. Additionally, the chi-square test was used for comparing categorical variables. Correlations were assessed using the Spearman rank correlation test. A p-value of less than 0.05 was regarded as statistically significant.

## Results

Adhering to the inclusion and exclusion criteria, the study was finalized with 71 patients with FMS and 70 healthy controls. The median ages of the FMS and control groups were 50 (31–72) and 49 (26–83), respectively. Of the FMS patients, 62 (87.3%) were female, whereas 60 (85.7%) of the healthy controls were female. No significant differences were observed in the essential characteristics of the groups (*p* > 0.05) (Table 1).

**Table 1:** The baseline features of the groups

	FMS group	Control group	*p*
Age^*^ (years)	50 (31–72)	49 (26–83)	0.347
BMI^*^ (kg/m²)	25.73 (17.96–33.33)	25.60 (20.22–34.92)	0.732
Sex (n) (%)			
Female	62 (87.3)	60 (85.7)	0.224
Male	9 (12.7)	10 (14.3)	
Educational status (n) (%)			
Primary School	19 (26.8)	16 (22.9)	
Middle School	8 (11.3)	7 (10)	0.201
High School	23 (32.4)	24 (34.3)	
University	18 (25.4)	19 (27.1)	
Higher than university	3 (4.1)	4 (5.7)	
Occupational status (n) (%)			
Working	34 (47.9)	36 (51.4)	0.101
Not working/Housewife	32 (45.1)	30 (42.9)	
Retired	5 (7)	4 (5.7)	

^*^: Data are expressed as median (minimum-maximum)

BMI: Body mass index; FMS: Fibromyalgia syndrome; kg: Kilogram; m²: Square meter; n: Number; %: Percentage

The median uncorrected visual acuity (decimals) for the right eye in the FMS and control groups were 0.80 (0.20–1) and 0.80 (0.40–1), respectively (*p* = 0.124). For the left eye, the values were 0.80 (0.20–1) and 0.80 (0.40–1) (*p* = 0.131). Furthermore, the median best-corrected visual acuity for the right eye (decimals) was 1 (0.20–1) in the FMS group and 1 (0.60–1) in the control group (*p* = 0.463). The median best-corrected visual acuity data for the left eye were 1 (0.25–1) and 1 (0.70–1) in the FMS and control groups (*p* = 0.132) (Table 2).

**Table 2:** Comparison of visual acuities between fibromyalgia and control groups

Parameter	FMS group	Control group	*p*
Uncorrected visual acuity right (decimals)	0.80 (0.20–1)	0.80 (0.40–1)	0.124
Uncorrected visual acuity left (decimals)	0.80 (0.20–1)	0.80 (0.40–1)	0.131
Best-corrected visual acuity right (decimals)	1 (0.20–1)	1 (0.60–1)	0.463
Best-corrected visual acuity left (decimals)	1 (0.25–1)	1 (0.70–1)	0.132

Data are expressed as median (minimum-maximum)

Comparing the FMS and control groups revealed no statistically significant differences in spherical equivalent (diopter), axis (degree), intraocular pressure (mmHg), and central corneal thickness (µm) for the right and left eyes (*p* > 0.05). The median astigmatism in the right eye (diopter) was − 0.50 (-8.25–0) for the FMS group and − 0.12 (-1.75–0.50) for the control group (*p* = 0.012). The left eye data were − 0.50 (-2–1.50) for the FMS group and 0 (-1.75–0.75) for the control group (*p* = 0.056) (Table 3).

**Table 3:** Comparison of spherical equivalent, astigmatism, axis, intraocular pressure, and central corneal thickness between fibromyalgia and control groups

Parameter	FMS group	Control group	*p*
Spherical equivalent-right (diopter)	0 (-3–6.75)	0 (-4.50–4.50)	0.683
Spherical equivalent-left (diopter)	0 (-4.50–2.50)	0 (-4.50–4.50)	0.474
Astigmatism-right (diopter)	-0.50 (-8.25–0)	-0.12 (-1.75–0.50)	0.012
Astigmatism-left (diopter)	-0.50 (-2–1.50)	0 (-1.75–0.75)	0.056
Axis-right (degree)	102.50 (4–180)	80 (5–180)	0.340
Axis-left (degree)	89 (5–175)	110 (2–180)	0.223
Intraocular pressure-right (mmHg)	15 (10–21)	14 (10–24)	0.142
Intraocular pressure-left (mmHg)	15 (9–25)	14.50 (10–21)	0.053
Central corneal thickness-right (µm)	530 (460–610)	530 (490–600)	0.570
Central corneal thickness-left (µm)	530 (470–610)	530 (500–600)	0.866

Data are expressed as median (minimum-maximum)

Meibomian gland dysfunction was identified in 26 (36.6%) individuals in the FMS group, whereas it was identified in 3 (4.3%) participants in the control group (*p* < 0.001). Dry eye disease was detected in 43 (60.6%) participants in the FMS group and 15 (21.4%) participants in the control group (*p* < 0.001). In the FMS group, 30 patients (69.8%) exhibited mild dry eye disease, whereas 12 individuals (80%) in the control group presented with mild dry eye disease. No statistically significant difference in dry eye severity was observed across the groups (*p* = 0.585) (Table 4).

**Table 4:** Comparison of meibomian gland dysfunction and dry eye disease between fibromyalgia and control groups

Parameter	FMS group	Control group	*p*
Meibomian gland dysfunction (*n*)			
-Yes	26 (36.6%)	3 (4.3%)	< 0.001
-No	45 (63.4%)	67 (95.7%)	
Dry eye disease (*n*)			
-Yes	43 (60.6%)	15 (21.4%)	< 0.001
-No	28 (39.4%)	55 (78.6%)	
Dry eye severity (n)			
- Mild	30 (69.8%)	12 (80%)	
- Moderate	11 (25.6%)	3 (20%)	0.585
- Severe	2 (4.6%)	0 (0%)	

No statistically significant correlation was detected between FIQ data and spherical equivalent (diopter), astigmatism (diopter), axis (degree), intraocular pressure (mmHg), and central corneal thickness (µm) for the right and left eyes in the patient group (*p* > 0.05).

## Discussion

The results demonstrated some ocular changes between FMS patients and controls. Notably, the FMS group had higher astigmatism, and meibomian gland dysfunction and dry eye disease were more frequent. The groups did not vary significantly regarding visual acuity, spherical equivalent, intraocular pressure, or central corneal thickness. Furthermore, the level of dry eye disease was not significantly distinct across the groups, and there was no correlation between FMS severity and ocular parameters.

No significant differences were observed between FMS patients and controls for visual acuity metrics, indicating that FMS does not directly affect visual clarity. Current findings suggest that FMS does not seem to impair the actual acuity of vision under standard assessment circumstances. Additionally, there was no statistically significant difference between the groups in terms of spherical equivalent, axis, intraocular pressure, or central corneal thickness. Schuster et al. [20] compared patients with FMS and those with non-FMS musculoskeletal pain, revealing no significant difference in visual acuity between the groups. These findings imply that, while FMS may be linked with ocular changes [7], it does not appear to significantly influence visual acuity under the settings investigated. Erkan Turan et al. [21] investigated ocular surface abnormalities in the corneal setting and discovered substantial differences in the FMS group. Some of the patients in this research were receiving FMS-related drugs. Including newly diagnosed patients who did not use FMS-related medication may explain the lack of significant differences in the parameters in our analysis.

Statistically significant differences in astigmatism were noted between the FMS and control groups for the right eye. This outcome may be related to dry eye disease, which is identified more frequently in the FMS group. Astigmatism and dry eye disease are frequently interrelated since the ocular surface abnormalities resulting from dry eye may affect astigmatism. In individuals with dry eyes, the tear film is often unstable or inadequate, resulting in an irregular moisture distribution on the corneal surface. This inconsistent tear film can affect the corneal shape, causing fluctuations in refractive power and raising the risk of astigmatism. Furthermore, dry eye symptoms involving irritation and inflammation can alter the stability of the corneal epithelium, which contributes to optical distortions [22, 23].

The frequency of dry eye disease and meibomian gland dysfunction was statistically significantly higher in FMS patients than in healthy controls. In addition to being a form of sebaceous gland, the meibomian gland is responsible for the secretion of meibum, a substance composed of polar and nonpolar lipids. Meibum is transported to the eye’s surface, covering the aqueous layer, maintaining the integrity of the tear film, and providing protection against organic materials and microorganisms. Meibomian gland dysfunction may result in a change in the composition of the tear film, pain in the eyelids and the eye, and evaporative dry eye, among other symptoms [24, 26].

In line with our findings, Türkyilmaz et al. [8] and Erkan Turan et al. [21] observed a higher frequency of dry eye disease among FMS patients. Several mechanisms may underlie these results. Initially, chronic pain in patients with FMS may be associated with dry eye disease. Hoffmann et al. [27] identified a correlation between chronic pain and dry eye disease in their meta-analysis. Irregular tear film and decreased tear production characteristics of dry eye disease are thought to be caused, in part, by inflammation, central sensitization, and dysregulation of the autonomic nervous system in FMS [7]. Although dry eye condition is minor clinically, subjective signs may be worsened by FMS’s central sensitization and elevated processing of sensory information.

The majority of FMS patients with dry eye disease were categorized as mild. No statistically significant difference in dry eye severity was detected between the FMS and control groups. No statistically significant association existed between FIQ and ophthalmological measures. The findings suggest FMS may elevate the risk of developing dry eye disease, although it does not inherently lead to more severe manifestations. FMS may trigger the development of dry eye disease, although the variables involved in pathogenesis do not increase the severity of dry eye disease above that seen in individuals in general. These findings highlight that dry eye disease may arise irrespective of overall fibromyalgia severity and may project regional or distinct systemic mechanisms instead of directly correlating with disease severity.

When considering these results, it is essential to consider several limitations. At this point, making any firm conclusions about cause and effect is impossible because the study employed a cross-sectional design. Finding temporal connections and causation requires longitudinal research. Secondly, there was a relatively small sample size. Future research should use larger samples to confirm these findings and reveal more nuanced distinctions. This research was conducted at a single tertiary care institution, which may introduce selection bias and limit the generalizability of our findings to the broader FMS population. Inflammatory rheumatic diseases were ruled out through verbal inquiry and examination of medical records. However, we did not conduct advanced immunological or rheumatological tests. This lack of assessments may limit our ability to conclusively exclude rheumatic disorders, an essential factor to consider when interpreting our findings. The study spanned three months, potentially failing to capture seasonal fluctuations in ocular symptoms, especially dry eye disease, due to the impact of environmental factors. Additionally, FMS patients, especially those undergoing treatment, may not have been able to benefit from the results because the study did not include participants who were taking medications linked to FMS to avoid confounding impacts. The individuals’ autonomic nervous system states, and the balance of their sympathetic and parasympathetic nervous systems was not evaluated. The participants did not undergo evaluation for factors associated with FMS, including sleep disruption, depression, and anxiety. Female participants made up the majority of the sample.

## Conclusion

Although visual acuity and key ocular metrics, including intraocular pressure, central corneal thickness, and spherical equivalent, were unaltered, FMS patients showed significantly higher frequencies of astigmatism, meibomian gland dysfunction, and dry eye disease. These data show that FMS may predispose to these issues. The increased frequency of dry eye disease and meibomian gland dysfunction in FMS patients underscores the necessity for regular ocular assessments in this patient group. Future research should prioritize longitudinal studies with larger sample sizes to ascertain causal links and investigate the temporal dynamics of ocular alterations in FMS. Furthermore, assessments of drug effects, autonomic function, and psychiatric abnormalities (such as depression, anxiety, and sleep issues) are crucial for clarifying the underlying mechanisms and pinpointing prospective treatment strategies.

## Electronic supplementary material

Below is the link to the electronic supplementary material.

Supplementary Material 1

Supplementary Material 2

Supplementary Material 3

Supplementary Material 4

## Data Availability

Raw data can be shared if requested.

## Declarations

### Disclaimer

No part of this article is copied, submitted, or published elsewhere in whole or in part in any language.
